# Alternatives in Assessing Mental Healthcare Disparities Using the Behavioral Risk Factor Surveillance System

**DOI:** 10.1089/heq.2017.0056

**Published:** 2018-08-01

**Authors:** Jin Liu, Ning Jiang, Amy Z. Fan, Rebecca Weissman

**Affiliations:** ^1^ Department of Educational Studies, College of Education, University of South Carolina, Columbia, South Carolina.; ^2^ National Institute on Alcohol Abuse and Alcoholism, National Institute of Health, Bethesda, Maryland.; ^3^ Department of Instruction and Teacher Education, College of Education, University of South Carolina, Columbia, South Carolina.

**Keywords:** diagnosis, mental healthcare disparities, missing data, subpopulation, treatment

## Abstract

**Purpose:** Identification of mental healthcare disparities through scales or questionnaires is an initial step to improve mental healthcare equity. This study was designed to investigate the performance of a single-item question relative to two psychiatric scales with multiple items in assessing mental healthcare disparities among U.S. adults using the Behavioral Risk Factor Surveillance System (BRFSS) data.

**Methods:** The current depression (CD) and serious psychological distress (SPD) were analyzed in 2010 and 2012 with the BRFSS using two multiple-item scales in selected states. Receipt of mental health diagnosis and mental health treatment was ascertained respectively. In both years, a single item of the number of mentally unhealthy days from Centers for Disease Control Health-Related Quality of Life-4 (CDC-HRQOL-4) core questions was used to ascertain frequent mental distress (FMD) in all states. Logistic regression was used to identify mental healthcare disparities among subpopulations. The *t*-test was used to analyze missing data patterns.

**Results:** Among adults who experienced FMD or CD in 2010, men, persons 65 years of age or older, non-Hispanic Blacks, persons who were currently or never married, and persons who were employed had a lower likelihood of receiving diagnosis of depression. Among adults who experienced FMD or SPD in 2012, men, persons 65 years of age or older, Hispanics and Blacks, persons who were currently employed or homemakers/students, and persons without healthcare coverage had a lower likelihood to receive mental health treatment. The missing rates of FMD were 1.8% (2010) and 1.4% (2012), while the missing rates of Patient Health Questionnaire 8 (PHQ-8) and Kessler 6 (K6) were 12.8% (2010) and 17.4% (2012). The samples with missing data were different from those without.

**Conclusions:** The single-item question is a valuable alternative in a large population surveillance to identify vulnerable subpopulations for lower mental health diagnosis and treatment.

## Introduction

Mental health and wellness have been widely stressed in the past decades and have received much attention in many studies.^1–4^ Research has shown that heart disease and depression are predicted to be the top two contributors to the global burden of disease by 2020.^5^ Data from the combined 2006 and 2008 Behavioral Risk Factor Surveillance System (BRFSS) indicated that about 9% of U.S. adults met criteria for current depression (CD) during the 2 weeks preceding the survey. Data from the 2015 National Survey on Drug Use and Health indicated that major depression during the past 12 months affected 6.7% (more than 16 million) of American adults.^6^

However, the proposed American Health Care Act would remove mental health as an essential healthcare benefit.^7^ More than half of adults with mental illness did not receive treatment.^8^ This is often due to barriers these adults experience in obtaining appropriate diagnosis and treatment for mental health issues, such as a shortage of mental health services, unawareness of problems and financial difficulty. Eliminating mental healthcare disparities and increasing diagnosis and treatment among all U.S. population segmentations have become important goals of the *Healthy People 2020* (www.healthypeople.gov).

Mental healthcare disparities, which are widely defined as a difference in mental healthcare accessibility and quality between different demographics, are common in the United States. Such disparities and the underlying risk factors are of concern to researchers, psychologists and healthcare professionals. A variety of risk factors have been proposed including socioeconomic status, racial/ethnicities, gender, and life events.^9–14^

The Agency for Healthcare Research and Quality (AHRQ)^15^ indicate that although overall healthcare quality is improving, disparities in service access have not improved significantly especially for people of color and low-income groups. Research suggests that minority patients, particularly African Americans, Hispanic Americans, and Asian or Pacific Islanders, have less access to mental healthcare services and are more likely to receive poor quality of mental health treatment in comparison to Caucasian patients.^16–19^ Socioeconomic status, as measured by education, income, or health insurance coverage, is another critical factor of mental healthcare disparities in the United States.^20,21^ Lack of health plan coverage and low education attainment are closely associated with lower mental healthcare utilization and quality. In addition, striking gender differences existed in utilization of mental healthcare services.^10,22^ Studies indicate that women tend to use more healthcare services and are more likely to report a prior diagnosis of mental health issues than men. Age and marital status may also influence the accessibility of mental healthcare services. For instance, adults 50 years or older were found to be less likely to visit mental health professionals^19^; married individuals may be more motivated to seek mental healthcare services.^23^

As the U.S. population continues to become more diverse, it takes substantial efforts to improve mental healthcare equity and eliminate disparities.^13^ Identification of mental healthcare disparities through population health surveillance is an initial step of the efforts.

The BRFSS is a state-based system of health surveys that collects information on health-risk behaviors, chronic health conditions, and use of healthcare services related to chronic disease and injury. Two optional modules (the Mental Illness and Stigma Module [MISM] and the Anxiety and Depression Module [ADM]) were added to the BRFSS since 2005. This allows an assessment of the prevalence of CD (from the Patient Health Questionnaire 8 [PHQ-8]) and Serious Psychological Distress (SPD from the Kessler 6 [K6]) in the U.S. population at large^24–26^ and relationship between mental health conditions.^27,28^ In addition, the Centers for Disease Control Health-Related Quality of Life-4 (CDC HRQOL-4) was part of the core questionnaire in the BRFSS and has been implemented in all states since 1993. One of the questions, “mentally unhealthy days during the past 30 days,” can be used to derive prevalence of frequent mental distress (FMD).

When using different modules in the BRFSS, states may be concerned whether the optional modules are useful or have additional values beyond core questions in assessing mental healthcare disparities in the general population and how to allocate limited surveillance resources to track prevalence and set health goals for prevention policy and program purpose. The length of a questionnaire was a concern as it was found to be negatively related to the response rate.^29^ More missing data are expected to occur in multiple-item psychiatric scales, such as the PHQ-8 and the K6, compared with a single item in the CDC HRQOL-4. Hence, this study was designed to compare the performance of the psychiatric scales (PHQ-8 and K6) relative to the single item in the CDC HRQOL-4 in assessing the mental healthcare disparities among U.S. subpopulations.

## Methods

The BRFSS is an ongoing, state-based, cross-sectional telephone health survey conducted by the random-digit dialing of noninstitutionalized U.S. adults.^30^ This survey collects data on sociodemographic information, chronic illness, health behaviors, access to healthcare, and other health-related information. Currently, The BRFSS collects data in all 50 states as well as the District of Columbia. The sample size is about 400,000 adults each year. Ethics approval is not applicable to the current study as this is a secondary database available online.

Before 2011, only landline data were collected. Poststratification weights were used to adjust for differences in selection probability and nonresponse, as well as partially nontelephone coverage. Since 2011, the BRFSS has been conducting both landline telephone- and cellular telephone-based surveys.^31^ Also starting in 2011, a new weighting methodology called iterative proportional fitting (or “raking”) replaced the poststratification method to weight the BRFSS data. The survey consists of three components: a core questionnaire, optional modules, and state-specific questions. The median survey response rate for all states and the District of Columbia, in 2012, was 45.2%, and ranged from 27.7% to 60.4%. The median survey response rate for all states and Washington DC, in 2010, was 54.6%, and ranged from 39.1% to 68.8%. The technical information, questionnaire, and survey data are available online at www.cdc.gov/brfss/data_documentation/index.htm.

### Measures

#### FMD from the CDC HRQOL-4

The CDC HRQOL-4 measures (also known as the Healthy Days measures) have demonstrated reliability and validity for population health surveillance (www.cdc.gov/hrqol/measurement.htm). The measures include four questions regarding self-rated health conditions and the number of recent days when physical health was impaired, mental health was impaired, and usual activities were limited due to poor physical or mental health. Specifically, impaired mental health was assessed by the question “Now thinking about your mental health, which includes stress, depression, and problems with emotions, for how many days during the past 30 days was your mental health not good?” A person who reported ≥14 days was identified as having FMD. This 14-day minimum period was selected because physicians and researchers often use a similar period as a marker for clinical depression and anxiety disorders.^32^ This module is one of the core sections within the BRFSS that has been implemented in all states since 1993.

#### CD from the PHQ-8 and diagnosis of depressive disorders

The ADM was implemented in 12 states in 2010, including Arizona, Georgia, Hawaii, Indiana, Louisiana, Mississippi, Missouri, Nevada, South Carolina, Vermont, Wisconsin, and Wyoming. The PHQ-8 is part of the ADM. The PHQ-8 is adapted from the PHQ-9.^33,34^ The PHQ-9 consists of the actual nine criteria for a diagnosis of depressive disorders, as defined in the Diagnostic and Statistical Manual for Mental Disorders, fourth edition (DSM-IV).^35^ The PHQ-9 has been validated among racially and ethnically diverse populations in primary care and for telephone administration.^36,37^ In the PHQ-8, the question assessing suicidal or self-injurious ideation from the PHQ-9 was eliminated. Research indicate that the PHQ-8 has operating characteristics similar to those of the PHQ-9 and is an acceptable alternative to PHQ-9.^33^

The response set of the PHQ-8 is standardized to be similar to the other BRFSS questions by asking the number of days in the previous 2 weeks the person had experienced a particular depressive symptom. This modification has been approved by the original authors of the PHQ-8. For analytic purposes, the modified response set can be converted back to the original response set: 0–1 days=“not at all,” 2–6 days=“several days,” 7–11 days=“more than half the days,” and 12–14 days=“nearly every day,” with 0–3 points assigned to the four categories, respectively. Item scores are summed for a total score of 0–24. A score from 0–4 represents no meaningful depressive symptoms; 5–9, mild depression; 10–14, moderate depression; 15–19, moderately severe depression; and 20–24, severe depression.^33^ For this study, the definition of CD is a total score of at least 10.^25^ About 88.0% of respondents gave complete answers to all eight items in the PHQ-8. One question of the ADM assesses doctor-diagnosed depressive disorders, this question asks, “Has a doctor or other healthcare provider EVER told you that you have a depressive disorder (including depression, major depression, dysthymia, or minor depression)?” The lifetime diagnosis of depression is positive to those who answered “yes” to the question.

#### SPD from the K6 and receipt of treatment

The K6 scale is part of the MISM in the BRFSS. The module was implemented in 10 states in 2012, including Illinois, Iowa, Minnesota, Missouri, Montana, Nevada, New Mexico, New York, Oregon, and Washington. The K6 is used primarily to screen for SPD.^38^ The K6 has been used in U.S. government health surveys in the United States and World Mental Health Surveys due to the brevity and strong psychometric properties.^39^

The K6 consists of six questions that ask respondents how frequently during the previous 30 days they felt: (1) “so sad that nothing could cheer … [them] up,” (2) “nervous,” (3) “restless or fidgety,” (4) “hopeless,” (5) “that everything was an effort,” and (6) “worthless.”

Respondents select one of the following occurrence frequency options—“all of the time,” “most of the time,” “some of the time,” “a little of the time,” and “none of the time.” Responses are scored on a 5-point Likert scale from 0 to 4 with lower scores indicating lower frequency of occurrence. Thus, the total scores range from 0 to 24 and respondents with a score of 13 or higher are considered to have SPD.^40^ One question which asked the respondent, “Are you now taking medicine or receiving treatment from a doctor or other health professional for any type of mental health condition or emotional problem?” is used at the end of the MISM to indicate the receipt of treatment for mental health conditions with the answer “yes.”

The following indices were used to diagnose mental healthcare disparities across demographic subgroups: proportion of recipients obtained diagnosis among adults who had FMD or CD and proportion of recipients received treatment among adults who had FMD or CD.

### Demographic characteristics

The following demographic variables were used to illustrate mental healthcare disparities: sex, age, race/ethnicity (Non-Hispanic white, non-Hispanic Blacks, Hispanics, or non-Hispanic others), education level (less than high school, high school graduates, college graduates, or higher), marital status (currently married/cohabiting, previously married, or never married), employment status (currently employed, currently unemployed, retired, unable to work, or homemaker/student), and health plan coverage (yes/no).

### Statistical analysis

SAS 9.4^41^ was used for data analysis. Strata, cluster, and weight variables were used to account for the complex sampling design of the BRFSS. Percentages and adjusted prevalence ratios (APR) for persons who obtained diagnosis of depression during their lifetime among people who had FMD and among people who had CD were compared using logistic regression side by side by selected sociodemographic characteristics. Similarly, percentages and APR for persons who received treatment among people who had FMD and among people who had SPD were compared using logistic regression side by side by selected sociodemographic characteristics. The default missing data technique (listwise deletion) was used. Finally, we discussed the missing data patterns in the PHQ-8 and the K6 and examined whether missing data would bias mental health assessment using *t*-tests.

## Results

### Disparities in diagnosis

The prevalence of CD in participating states in 2010 was 9.8% (standard error [SE]=0.2%), whereas prevalence of FMD in 2010 was 11.0% (SE=0.2%). The diagnosis rates among adults who experienced FMD or CD in 2010 were significantly different in association with sex, age, race/ethnicity, marital status, and employment status (Table 1). Among adults who experienced FMD or CD (symptom-positive) in 2010, the following subpopulations had a lower likelihood of obtaining diagnosis of depression: men, persons 65 years or older (vs. those ages 45–54 years), and non-Hispanic Blacks (vs. non-Hispanic whites) (Table 1). The following symptom-positive subpopulations were more likely to obtain a diagnosis of depression: previously married (vs. currently married), and those who were retired or unable to work (vs. currently employed). The overall diagnosis disparity patterns were similar across all demographic variables although some subgroups may reach statistical significance for one symptom measure but not for another measure. In addition, CD failed to detect any significant diagnosis disparities in association with education attainment. CD was unable to inform whether those symptom-positive individuals who were “currently unemployed” or “homemaker or student” would be more likely to receive depression diagnosis than those “employed.” On the other hand, CD was able to inform that all minority groups with symptom-positive individuals were less likely to obtain depression diagnosis than non-Hispanic whites; FMD only identified non-Hispanic Blacks to be the sole group who were significantly less likely to be diagnosed than non-Hispanic whites.

**Table 1.** **Percentage and Adjusted Prevalence Ratios for Persons Who Have Obtained Diagnosis of Depression During Their Lifetime Among Symptom-Positive Populations by Selected Sociodemographic Characteristics, 2010 Behavioral Risk Factor Surveillance System**

	Obtained diagnosis of depression among U.S. adults who have FMD		Obtained diagnosis of depression among U.S. adults who have CD	
	% (SE)	APR (95% CI)	% (SE)	APR (95% CI)
Sample size	7945		6545	
Total	51.4 (1.1)		56.6 (1.2)	
Sex				
Male	44.8 (1.9)	**0.61 (0.50–0.75)**	50.3 (2.1)	**0.64 (0.51–0.79)**
Female	55.8 (1.3)	1.00	60.4 (1.4)	1.00
*p* for difference	<0.001		<0.001	
Age				
18–24	42.8 (5.1)	0.72 (0.44–1.18)	42.6 (4.7)	0.83 (0.51–1.35)
25–34	46.9 (3.2)	0.96 (0.69–1.34)	54.1 (3.3)	1.26 (0.89–1.78)
35–44	50.6 (2.6)	0.95 (0.72–1.24)	57.7 (2.6)	1.07 (0.80–1.43)
45–54	57.6 (2.0)	1.00	62.2 (2.1)	1.00
55–64	60.1 (1.7)	0.94 (0.74–1.20)	64.5 (1.8)	0.88 (0.68–1.14)
65+	41.4 (1.8)	**0.40 (0.30–0.54)**	45.4 (2.2)	**0.33 (0.24–0.45)**
*p* for difference	<0.001		<0.001	
Race/ethnicity				
White non-Hispanic	55.1 (1.3)	1.00	61.1 (1.4)	1.00
Black non-Hispanic	37.1 (2.5)	**0.36 (0.28–0.46)**	43.8 (2.7)	**0.41 (0.31–0.54)**
Hispanic	45.0 (5.5)	0.69 (0.44–1.11)	45.1 (5.5)	**0.51 (0.32–0.79)**
Other, non-Hispanic	50.2 (3.8)	0.70 (0.48–1.10)	52.2 (4.6)	**0.61 (0.41–0.92)**
*p* for difference	<0.001		<0.001	
Education				
Less than high school	53.7 (2.8)	0.83 (0.62–1.11)	55.6 (2.7)	0.83 (0.62–1.10)
High school	48.4 (1.8)	**0.74 (0.60–0.91)**	54.5 (1.9)	0.85 (0.68–1.07)
Some college or higher	52.6 (1.6)	**1.00**	58.3 (1.8)	1.00
*p* for difference	0.13		0.33	
Marital status				
Currently married	48.8 (1.5)	1.00	55.3 (1.7)	1.00
Previously married	58.9 (1.7)	**1.37 (1.12–1.68)**	66.3 (1.7)	**1.51 (1.22–1.87)**
Never married	48.6 (2.8)	1.25 (0.91–1.72)	48.9 (2.7)	0.97 (0.71–1.32)
*p* for difference	<0.001		<0.001	
Employment status				
Currently employed	39.9 (1.7)	1.00	48.5 (2.0)	1.00
Currently unemployed	49.4 (3.2)	**1.73 (1.24–2.40)**	49.6 (3.4)	1.30 (0.93–1.83)
Retired	46.4 (2.0)	**2.29 (1.72–2.04)**	51.4 (2.4)	**2.28 (1.65–3.15)**
Unable to work	77.5 (1.6)	**6.27 (4.86–8.09)**	78.2 (1.4)	**4.59 (3.52–5.98)**
Homemaker, student	50.5 (3.9)	**1.63 (1.15–2.33)**	52.2 (3.7)	1.31 (0.93–1.96)
*p* for difference	<0.001		<0.001	
Health plan coverage				
Yes	52.1 (1.2)	1.00	58.4 (1.3)	1.00
No	49.7 (2.4)	0.97 (0.76–1.24)	51.7 (2.5)	0.86 (0.67–1.10)
*p* for difference	0.37		0.02	

Bold values: subgroups that had a significantly lower or higher likelihood of obtaining diagnosis of depression.

APR, adjusted prevalence ratio; CD, current depression; CI, confidence interval; FMD, frequent mental distress; SE, standard error.

### Disparities in treatment

The prevalence of SPD in participating states in 2012 was 3.9% (SE=0.2%), while prevalence of FMD in 2012 was 11.1% (SE=0.2%). The treatment rates among adults who experienced FMD or SPD in 2012 were significantly different in association with sex, age, race/ethnicity, education attainment, marital status, employment status, and health plan coverage (Table 2). Among adults who experienced FMD or SPD in 2012, the following subpopulations were less likely to receive treatment: men, persons 65 years or older (vs. those ages 45–54 years), non-Hispanic Blacks and Hispanics (vs. non-Hispanic whites), and persons who had no health plan coverage (Table 2). The following subpopulations with positive symptoms were more likely to receive treatments: currently unemployed, retired, and those unable to work (vs. those currently employed). Some discrepancies were noted. FMD was able to detect treatment disparities of individuals in different education attainment categories compared with SPD. FMD could inform that all racial minority groups were less likely to receive treatments than non-Hispanic whites while SPD could not.

**Table 2.** **Percentage (Standard Error) and Adjusted Prevalence Ratios (95% Confidence Interval) for Persons Who Have Used Medicine or Received Treatment Among Symptom-Positive Populations by Selected Sociodemographic Characteristics, 2012 Behavioral Risk Factor Surveillance System**

	Used medication or received treatment among U.S. adults who have FMD		Used medication or received treatment among U.S. adults who have SPD	
	% (SE)	APR (95% CI)	% (SE)	APR (95% CI)
Sample size	7033		2289	
Percentage	39.0 (1.1)		50.3 (2.2)	
Sex				
Male	30.5 (1.7)	**0.54 (0.44–0.66)**	37.9 (3.2)	**0.36 (0.26–0.51)**
Female	44.9 (1.4)	1.00	58.9 (2.8)	1.00
*p* for difference	<0.001		<0.001	
Age				
18–24	29.6 (3.9)	0.71 (0.43–1.16)	39.2 (7.7)	0.69 (0.32–1.49)
25–34	32.3 (2.7)	0.84 (0.59–1.20)	42.8 (5.9)	1.05 (0.57–1.92)
35–44	41.3 (2.6)	1.10 (0.82–1.48)	48.0 (5.4)	0.89 (0.50–1.58)
45–54	45.4 (2.2)	1.00	57.1 (3.9)	1.00
55–64	43.6 (2.2)	0.85 (0.65–1.11)	61.3 (3.9)	1.09 (0.67–1.77)
65+	38.9 (2.2)	**0.56 (0.39–0.81)**	49.9 (4.6)	**0.42 (0.20–0.85)**
*p* for difference	<0.001		0.02	
Race/ethnicity				
White non-Hispanic	43.0 (1.3)	1.00	55.1 (2.4)	1.00
Black non-Hispanic	25.8 (4.1)	**0.34 (0.22–0.54)**	44.4 (9.5)	**0.41 (0.18–0.94)**
Hispanic	28.9 (3.5)	**0.66 (0.44–1.00)**	28.9 (5.1)	**0.43 (0.24–0.77)**
Other, non-Hispanic	33.5 (3.5)	**0.67 (0.47–0.96)**	56.1 (6.2)	1.05 (0.57–1.95)
*p* for difference	<0.001		0.01	
Education				
Less than high school	34.5 (3.2)	**0.66 (0.44–1.00)**	41.2 (5.3)	0.50 (0.31–0.82)
High school	37.3 (1.8)	**0.67 (0.47–0.96)**	49.6 (3.4)	0.78 (0.54–1.12)
Some college or higher	41.7 (1.4)	1.00	57.8 (2.7)	1.00
*p* for difference	0.05		0.01	
Marital status				
Currently married	39.2 (1.6)	1.00	57.1 (3.2)	1.00
Previously married	43.7 (1.8)	1.17 (0.94–1.45)	49.6 (4.0)	0.72 (0.47–1.09)
Never married	34.9 (2.2)	1.15 (0.87–1.53)	46.1 (3.9)	0.87 (0.53–1.43)
*p* for difference	0.001		0.07	
Employment status				
Currently employed	29.3 (1.5)	1.00	37.4 (3.7)	1.00
Currently unemployed	36.6 (3.1)	**1.97 (1.44–2.70)**	44.3 (4.4)	**1.73 (1.05–2.84)**
Retired	41.6 (2.3)	**2.04 (1.46–2.86)**	57.1 (4.6)	**2.86 (1.39–5.87)**
Unable to work	59.7 (2.9)	**4.00 (3.00–5.32)**	63.3 (4.2)	**2.83 (1.78–4.50)**
Homemaker, student	33.0 (3.1)	1.26 (0.90–1.76)	43.1 (6.7)	1.20 (0.66–2.2)
*p* for difference	<0.001		<0.001	
Health plan coverage				
Yes	43.7 (1.3)	1.00	60.1 (2.6)	1.00
No	24.3 (2.1)	**0.45 (0.34–0.60)**	29.1 (3.6)	**0.31 (0.21–0.45)**
*p* for difference	<0.001		<0.001	

Bold values: subgroups that had a significantly lower or higher likelihood of obtaining diagnosis of depression.

SPD, serious psychological distress.

### Pattern of missing data and its influence

In 2010, of 12.8% of respondents who had missing data in the PHQ-8, about 4.7% of the participants skipped the entire scale and 8.1% did not give valid answers to at least one question. In 2012, among the 17.4% of respondents who had missing data in the K6, around 15.5% of the respondents skipped the entire scale and around 1.9% of the respondents did not give valid answers to at least one question.

Additionally, respondents who completed the PHQ-8 had an average of 3.4 mentally unhealthy days, while respondents who did not complete the PHQ-8 had an average of 4.5 mentally unhealthy days (*t*=5.67, *p*<0.001). In other words, mental health burden or prevalence of CD may have been underestimated in 2010 by the PHQ-8. Respondents who completed the K6 had an average of 3.4 mentally unhealthy days while individuals who did not complete the K6 had an average of 3.2 mentally unhealthy days (*t*=2.51, *p*=0.01). Although the difference was trivial, the significant discrepancy still indicated that missing data may bias the estimates of the mental health burden to some extent. In contrast, the missing rates of FMD were much lower (1.8% in 2010 and 1.4% in 2012).

## Discussion

Results from this study were consistent with previous studies' findings that mental healthcare disparities existed among different U.S. demographics.^19,21–23^ The AHRQ identified the racial minority groups and older adults as priority populations in healthcare.^42^ Our findings echoed with these recommendations since indeed individuals in these groups were less likely to be diagnosed or treated for mental health problems. Attention should also be paid to lower-educated, currently married, and currently employed individuals who have mental health problems. These individuals were at a higher risk of underdiagnosis and undertreatment. Lack of health plan coverage was a significant deterrent for treatment but not for diagnosis. Most impressively, the results from the BRFSS data indicated that a single question from the HRQOL-4 had compatible capacity on identifying mental healthcare disparities in subpopulations with multi-item psychiatric scales (i.e., the PHQ-8 and the K6).

Multi-item psychiatric scales had been widely adopted in other population-based surveys as well. For example, the K6 was adopted in the National Health Interview Survey^24^ and National Survey on Drug Use and Health,^43^ and the PHQ-9 in the National Health and Nutrition Examination Survey.^24^ Although using a well-validated psychiatric scale is useful for monitoring trends and making comparisons between different demographics, administering such scales to general populations in public health surveillance may be burdensome.

Missing data occur in survey research for a variety of reasons and are a cause of concern for survey analysis.^44^ Survey fatigue may occur as a result of a lengthy survey and negatively affect the quality and validity. The length of the questionnaire is negatively related to the response rate.^29,45,46^ While only less than 2% of the respondents were missing for the question regarding mentally unhealthy days in 2010 and 2012, over 10% of the respondents failed to give valid information for all items used to screen CD or SPD. Rates of less than 1% missing data are considered trivial, 1–5% are manageable; however, 5–15% missing data require sophisticated methods to handle, and more than 15% may severely impact any kind of interpretation.^47^ Additionally, nonresponses may affect the quality of the estimates when the respondents and nonrespondents exhibit different characteristics with respect to the study variables.^48^ Individuals who completed the PHQ-8 or the K6 had significantly different number of mentally unhealthy days compared with those who did not complete the PHQ-8 or the K6 in the current study.

To compose a parsimonious set of questions in assessing mental healthcare disparities in the general population, we would recommend the following: (1) Have you experienced mentally unhealthy days during the past 30 days? (2) Have you received any diagnosis of mental disorders (over the course of your lifetime and during the past year)? A complete set of choices and “check all that apply” should be given in the instruction; (3) Have you received any treatment of mental disorders? All kind of possible treatment modalities should be listed for the respondents to “check all that apply.”

The study was not intended to nullify the value of the PHQ-8 or the K6 completely in general health surveillance. It demonstrated that the PHQ-8 and the K6 can indeed be used to identify healthcare disparity. The consistent pattern/tendency but some nonsignificant results compared with FMD were mostly due to the reduced power caused by lower sample size due to lower prevalence of SPD than FMD within the general population. Although the CDC HRQOL-4 was retained in the BRFSS core questionnaire, the ADM and MISM were administered in alternative years by states voluntarily. Thus, any linkage between diagnosis and treatment are impossible in the BRFSS for a certain year. We are unable to make inferences on ratios of treatment to diagnosis, which may be indicative of mental healthcare disparities as well.

The single-item (i.e., mentally unhealthy days) performed equally well as the multi-item psychiatric scales in assessing mental healthcare disparities in the BRFSS. States can use greater scrutiny to adopt multi-item psychiatric scales or only use the CDC HRQOL-4 together with questions on diagnosis and treatment to detect mental healthcare disparities among their state population.

## Abbreviations Used

ADM: Anxiety and Depression Module
AHRQ: Agency for Healthcare Research and Quality
APR: adjusted prevalence ratio
BRFSS: Behavioral Risk Factor Surveillance System
CD: current depression
CDC-HRQOL-4: Centers for Disease Control Health-Related Quality of Life-4
FMD: frequent mental distress
K6: Kessler 6
MISM: Mental Illness and Stigma Module
PHQ: Patient Health Questionnaire
SE: standard error
SPD: serious psychological distress
